# *catena*-Poly[copper(II)-bis­(μ-2-ethyl-5-methyl­imidazole-4-sulfonato-κ^3^ *N* ^3^,*O* ^4^:*O* ^4′^)]

**DOI:** 10.1107/S160053681103409X

**Published:** 2011-08-27

**Authors:** Andrew P. Purdy, Ray J. Butcher

**Affiliations:** a Chemistry Division, Code 6120 Naval Research Laboratory, 4555 Overlook Avenue SW, Washington, DC 20375, USA; b Department of Chemistry, Howard University, 525 College Street NW, Washington, DC 20059, USA

## Abstract

In the title compound, [Cu(C_6_H_9_N_2_O_3_S)_2_]_*n*_, the copper(II) ion sits on an inversion center and is chelated by the imidazole N and sulfonate O atoms of two ligands in equatorial positions. O atoms of adjacent mol­ecules coordinate in the axial positions. Jahn–Teller tetra­gonal distortion is evident in the coordination geometry [Cu—N and Cu—O equatorial distances of 1.971 (3) and 2.045 (2) Å, respectively, with a Cu—O axial distance of 2.433 (3) Å]. The structure is propagated by an infinite chain of eight-membered (Cu—O—S—O)_2_ ring systems along the *a* axis. Only N—H⋯O hydrogen bonding exists between the chains.

## Related literature

For literature related to the 2-ethyl-4-methyl­imidazole-5-sulfonic acid ligand, see: Purdy *et al.* (2007 ▶). For sulfonate-bridged Cu complexes with Cu–sulfonate chains, see: van Albada *et al.* (2001 ▶); Cai *et al.* (2004 ▶); Doyle *et al.* (1983 ▶); Han *et al.* (2006 ▶); He *et al.* (2009 ▶); Hubig *et al.* (2000 ▶); Sreenivasulu *et al.* (2005 ▶); Timmermans *et al.* (1984 ▶). For geometric data, see: Jahn & Teller (1937 ▶). 
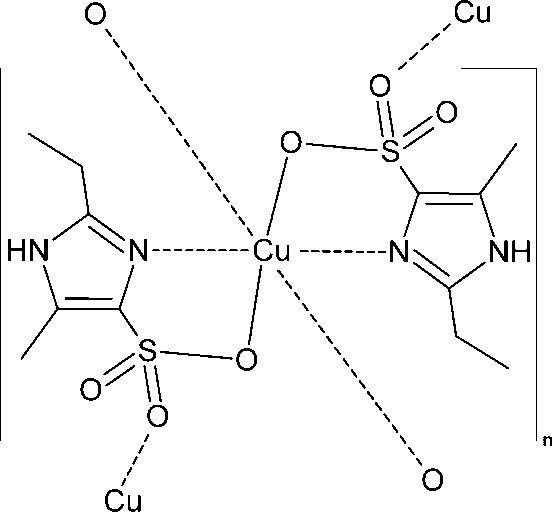


## Experimental

### 

#### Crystal data

[Cu(C_6_H_9_N_2_O_3_S)_2_]
*M* *_r_* = 441.96
Monoclinic, 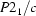

*a* = 5.0732 (4) Å
*b* = 11.8367 (10) Å
*c* = 13.6810 (11) Å
β = 94.473 (7)°
*V* = 819.04 (12) Å^3^
*Z* = 2
Cu *K*α radiation
μ = 4.64 mm^−1^
*T* = 295 K
0.44 × 0.32 × 0.24 mm

#### Data collection

Oxford Diffraction Xcalibur Ruby Gemini diffractometer
Absorption correction: analytical [*CrysAlis PRO* (Oxford Diffraction, 2007 ▶) based on expressions derived by Clark & Reid (1995 ▶)*T*_min_ = 0.270, *T*_max_ = 0.445
3114 measured reflections
1704 independent reflections
1605 reflections with *I* > 2σ(*I*)
*R*_int_ = 0.033

#### Refinement

*R*[*F* ^2^ > 2σ(*F* ^2^)] = 0.064
*wR*(*F* ^2^) = 0.181
*S* = 1.08
1704 reflections
117 parameters
H-atom parameters constrained
Δρ_max_ = 1.15 e Å^−3^
Δρ_min_ = −0.84 e Å^−3^

### 

Data collection: *CrysAlis PRO* (Oxford Diffraction, 2007 ▶); cell refinement: *CrysAlis PRO*; data reduction: *CrysAlis PRO*; program(s) used to solve structure: *SHELXS97* (Sheldrick, 2008 ▶); program(s) used to refine structure: *SHELXL97* (Sheldrick, 2008 ▶); molecular graphics: *SHELXTL* (Sheldrick, 2008 ▶); software used to prepare material for publication: *SHELXTL*.

## Supplementary Material

Crystal structure: contains datablock(s) I, global. DOI: 10.1107/S160053681103409X/om2453sup1.cif

Structure factors: contains datablock(s) I. DOI: 10.1107/S160053681103409X/om2453Isup2.hkl

Additional supplementary materials:  crystallographic information; 3D view; checkCIF report

## Figures and Tables

**Table 1:** Selected geometric parameters (Å, °)

Cu1—N1	1.971 (3)
Cu1—O1	2.045 (2)
Cu1—O1^i^	2.045 (2)
Cu1—O3^ii^	2.433 (3)

Symmetry codes: (i) 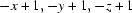; (ii) 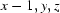.

**Table 2:** Hydrogen-bond geometry (Å, °)

*D*—H⋯*A*	*D*—H	H⋯*A*	*D*⋯*A*	*D*—H⋯*A*
N2—H2*C*⋯O2^iii^	0.86	1.95	2.784 (4)	164

Symmetry code: (iii) 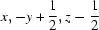.

**Fig. 1.**
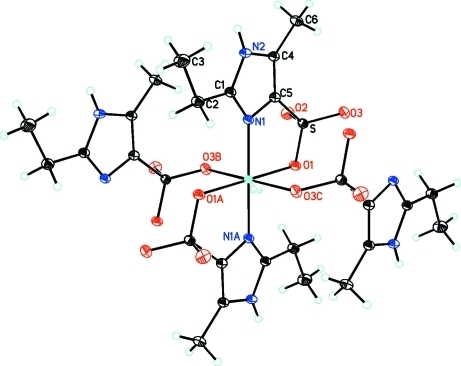
Diagram of C12H18CuN4O6S2 illustrating the atom numbering scheme used. Thermal displacement parameters are at the 30% probability level. [symmetry codes for labelled atoms: O1A, N1A, 1 - x, 1 - y, 1 - z; O3B, x - 1, y, z; O3C, 2 - x, 1 - y, 1 - z]

**Fig. 2.**
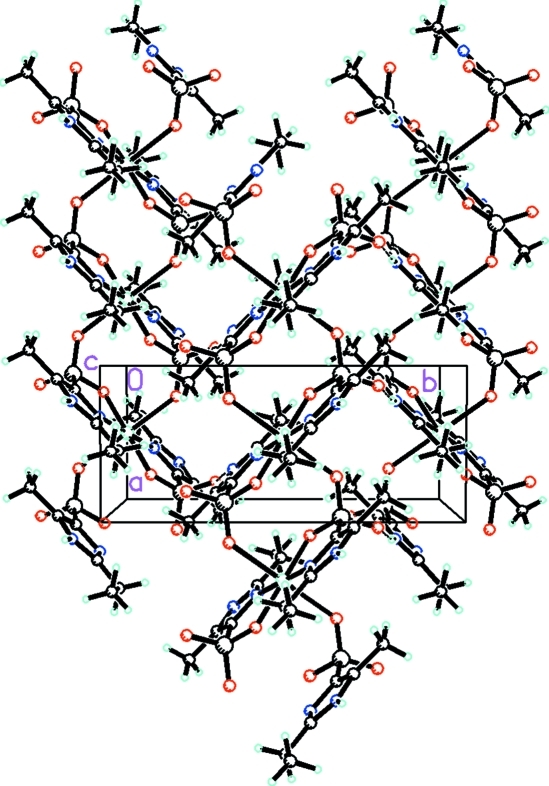
The molecular packing for C12H18CuN4O6S2 viewed down the c axis.

## supplementary crystallographic information

### Comment

In the title compound, the copper(II) ion sits on an inversion center and is chelated by the imidazole N1 and sulfonate O1 of two ligands. The two chelate rings on a Cu are 5-membered and co-planar. Two O3 O atoms of adjacent molecules coordinate the axial positions with the usual Jahn-Teller tetragonal distortion (Jahn & Teller, 1937) ((Cu—N and Cu—O equatorial distances of 1.971 (3) and 2.045 (2) Å, respectively, with a Cu—O axial distance of 2.433 (3) Å) and link the Cu atoms in an infinite chain of 8-membered (Cu—O—S—O)_2_ rings along the *a* axis. A number of examples exist for catenated 8-membered rings of sulfonate bridged copper(I) ions - Doyle *et al.* (1983), Han *et al.* (2006), Timmermans *et al.* (1984), and Hubig *et al.* (2000). All previous examples have 4 or 5 coordinate copper(I) and edge-shared catenation between the rings. This infinite chain of (Cu—O—S—O)_2_ rings is unique for copper(II), and its rings are corner shared and linear. The Cu coordination is nearly octahedral, with adjacent angles ranging from 84.92 (10) to 93.10 (10)°. Our silver(I) complex of the same ligand (Purdy, *et al.* (2007)) has edge shared 8-membered rings connected by a tetrahedral Ag atom. The CuO_4_ moieties are planar, and are nearly perpendicular (85.00 (8)°) to a plane composed of the linear N—Cu—N units within a chain. Likewise the plane formed by the 5-membered Cu—N—C—S—O chelate rings forms a dihedral angle of 86.61 (8)° with the plane formed by the Cu and O3 atoms within a chain.

As noted above, although all Cu—O distances are within the ranges normally observed in sulfonate complexes, the Cu—O3 distance is 0.4 Å longer than Cu—O1 as is seen in bis(µ_2_-(2-((2-oxybenzylidene)amino)ethyl)sulfonato)-diaqua-dicopper(II) dihydrate and other similar chelated copper(II) sulfonates (Sreenivasulu *et al.*, 2005; Cai *et al.*, 2004). Copper(II) sulfonates where the sulfonate is not part of a chelate ring tend to have Cu—O distance of 2.3 Å or greater as for example in bis(µ_2_-hydroxo)-bis(µ_2_-trifluoromethanesulfonato-*O*,*O*')-bis(4,4- dimethyl-2,2'-bipyridine)-di-copper(II) (van Albada, *et al.*, 2001) and in a sulfonate bridged complex (He, *et al.*, 2009).

The O2 atoms of the sulfonates are hydrogen bonded to the hydogen on N2 of the imidazole ring of an adjacent chain, at a N—O distance of 2.784 (4) Å. This interaction bonds the chains into a fully three-dimensional structure.

### Experimental

Both 1:1 and 1:1.5 solutions of the potassium salts of the 2-ethyl-4-methyl-imidazole-5-sulfonic acid were prepared by combining 1 g (5.25 mmol) of the free acid with 1 and 1.5 equivalents of KOH solution respectively, and diluting the solutions to 1*M* based on K^+^. (All solutions were made with distilled water.) Two test reactions were done in vials with a 1*M* solution of CuCl_2_.2H_2_O, and a 0.2 ml metered pipet was used for the additions. In reaction #1, 0.2 ml of CuCl_2_ solution was combined with 0.4 ml of the 1:1 solution. In reaction #2, 0.4 ml or the CuCl_2_ solution was combined with 0.6 ml of the 1:1.5 solution. Both solutions were heated to a boil and allowed to cool. Both reactions produced a pale green precipitate, but green crystals of the title compound grow in #2 only, over several days.

### Refinement

H atoms were placed in geometrically idealized positions and constrained to ride on their parent atoms with an N—H distance of 0.86 Å and C—H distances of 0.97 Å*U*_iso_(H) = 1.2*U*_eq_(C) and 0.96 Å for CH_3_ [*U*_iso_(H) = 1.5*U*_eq_(C)].

### Crystal data

**Table:**

[Cu(C_6_H_9_N_2_O_3_S)_2_]	*F*(000) = 454
*M**_r_* = 441.96	*D*_x_ = 1.792 Mg m^−^^3^
Monoclinic, *P*2_1_/*c*	Cu *K*α radiation, λ = 1.54178 Å
Hall symbol: -P 2ybc	Cell parameters from 2637 reflections
*a* = 5.0732 (4) Å	θ = 5.0–77.1°
*b* = 11.8367 (10) Å	µ = 4.64 mm^−^^1^
*c* = 13.6810 (11) Å	*T* = 295 K
β = 94.473 (7)°	Chunk, pale green-blue
*V* = 819.04 (12) Å^3^	0.44 × 0.32 × 0.24 mm
*Z* = 2	

### Data collection

**Table:**

Oxford Diffraction Xcalibur Ruby Gemini diffractometer	1704 independent reflections
Radiation source: Enhance (Cu) X-ray sealed tube	1605 reflections with *I* > 2σ(*I*)
graphite	*R*_int_ = 0.033
Detector resolution: 10.51 pixels mm^-1^	θ_max_ = 77.6°, θ_min_ = 5.0°
ω scans	*h* = −6→6
Absorption correction: analytical (*CrysAlis PRO*; Oxford Diffraction, 2007; Clark & Reid, 1995)'	*k* = −14→14
*T*_min_ = 0.270, *T*_max_ = 0.445	*l* = −17→10
3114 measured reflections	

### Refinement

**Table:**

Refinement on *F*^2^	Primary atom site location: structure-invariant direct methods
Least-squares matrix: full	Secondary atom site location: difference Fourier map
*R*[*F*^2^ > 2σ(*F*^2^)] = 0.064	Hydrogen site location: inferred from neighbouring sites
*wR*(*F*^2^) = 0.181	H-atom parameters constrained
*S* = 1.08	*w* = 1/[σ^2^(*F*_o_^2^) + (0.1339*P*)^2^ + 0.7529*P*] where *P* = (*F*_o_^2^ + 2*F*_c_^2^)/3
1704 reflections	(Δ/σ)_max_ < 0.001
117 parameters	Δρ_max_ = 1.15 e Å^−^^3^
0 restraints	Δρ_min_ = −0.84 e Å^−^^3^

### Special details

**Table:**

Geometry. All e.s.d.'s (except the e.s.d. in the dihedral angle between two l.s. planes) are estimated using the full covariance matrix. The cell e.s.d.'s are taken into account individually in the estimation of e.s.d.'s in distances, angles and torsion angles; correlations between e.s.d.'s in cell parameters are only used when they are defined by crystal symmetry. An approximate (isotropic) treatment of cell e.s.d.'s is used for estimating e.s.d.'s involving l.s. planes.
Refinement. Refinement of *F*^2^ against ALL reflections. The weighted *R*-factor *wR* and goodness of fit *S* are based on *F*^2^, conventional *R*-factors *R* are based on *F*, with *F* set to zero for negative *F*^2^. The threshold expression of *F*^2^ > σ(*F*^2^) is used only for calculating *R*-factors(gt) *etc*. and is not relevant to the choice of reflections for refinement. *R*-factors based on *F*^2^ are statistically about twice as large as those based on *F*, and *R*- factors based on ALL data will be even larger.

### Fractional atomic coordinates and isotropic or equivalent isotropic displacement parameters (Å^2^)

**Table:**

	*x*	*y*	*z*	*U*_iso_*/*U*_eq_	
Cu1	0.5000	0.5000	0.5000	0.0255 (3)	
S1	0.94199 (14)	0.32615 (6)	0.53104 (5)	0.0252 (3)	
O1	0.8041 (5)	0.4192 (2)	0.57814 (17)	0.0327 (6)	
O2	0.8628 (6)	0.2154 (2)	0.56252 (19)	0.0402 (7)	
O3	1.2247 (5)	0.3403 (2)	0.53856 (19)	0.0345 (6)	
N1	0.6262 (5)	0.4204 (2)	0.38608 (19)	0.0251 (6)	
N2	0.7482 (6)	0.3464 (2)	0.2509 (2)	0.0283 (6)	
H2C	0.7537	0.3326	0.1894	0.034*	
C1	0.5837 (6)	0.4210 (3)	0.2898 (2)	0.0257 (6)	
C2	0.3917 (8)	0.4933 (3)	0.2308 (3)	0.0316 (8)	
H2A	0.4281	0.5717	0.2474	0.038*	
H2B	0.2151	0.4763	0.2492	0.038*	
C3	0.3959 (10)	0.4793 (4)	0.1198 (3)	0.0466 (10)	
H3A	0.2724	0.5309	0.0872	0.070*	
H3B	0.3475	0.4032	0.1018	0.070*	
H3C	0.5704	0.4949	0.1008	0.070*	
C4	0.9057 (7)	0.2957 (3)	0.3252 (2)	0.0272 (6)	
C5	0.8264 (7)	0.3429 (3)	0.4086 (2)	0.0263 (6)	
C6	1.1174 (8)	0.2112 (3)	0.3088 (3)	0.0375 (8)	
H6A	1.2247	0.1995	0.3689	0.056*	
H6B	1.2257	0.2389	0.2595	0.056*	
H6C	1.0376	0.1410	0.2875	0.056*	

### Atomic displacement parameters (Å^2^)

**Table:**

	*U*^11^	*U*^22^	*U*^33^	*U*^12^	*U*^13^	*U*^23^
Cu1	0.0248 (4)	0.0294 (5)	0.0230 (4)	0.0050 (2)	0.0057 (3)	−0.0033 (2)
S1	0.0256 (5)	0.0264 (5)	0.0241 (5)	0.0014 (3)	0.0055 (3)	0.0015 (2)
O1	0.0322 (12)	0.0400 (13)	0.0261 (11)	0.0090 (10)	0.0036 (9)	−0.0048 (9)
O2	0.0507 (16)	0.0371 (15)	0.0333 (13)	−0.0043 (12)	0.0073 (11)	0.0076 (10)
O3	0.0269 (12)	0.0368 (13)	0.0401 (14)	0.0023 (9)	0.0048 (10)	0.0012 (10)
N1	0.0245 (12)	0.0257 (13)	0.0258 (13)	0.0007 (10)	0.0066 (10)	−0.0016 (9)
N2	0.0323 (15)	0.0299 (13)	0.0235 (12)	0.0021 (11)	0.0079 (11)	−0.0032 (10)
C1	0.0267 (14)	0.0240 (14)	0.0273 (15)	−0.0009 (11)	0.0070 (11)	−0.0017 (11)
C2	0.0343 (18)	0.0326 (17)	0.0282 (17)	0.0050 (12)	0.0045 (14)	0.0017 (12)
C3	0.059 (3)	0.053 (2)	0.0269 (17)	0.012 (2)	0.0002 (17)	0.0004 (16)
C4	0.0287 (15)	0.0269 (14)	0.0269 (14)	0.0012 (12)	0.0071 (12)	−0.0021 (12)
C5	0.0293 (15)	0.0245 (14)	0.0256 (14)	0.0011 (12)	0.0050 (12)	0.0002 (11)
C6	0.0361 (18)	0.0340 (18)	0.0433 (19)	0.0102 (15)	0.0081 (15)	−0.0062 (15)

### Geometric parameters (Å, °)

**Table:**

Cu1—N1^i^	1.971 (3)	N2—C4	1.380 (4)
Cu1—N1	1.971 (3)	N2—H2C	0.8600
Cu1—O1	2.045 (2)	C1—C2	1.487 (5)
Cu1—O1^i^	2.045 (2)	C2—C3	1.529 (5)
Cu1—O3^ii^	2.433 (3)	C2—H2A	0.9700
Cu1—O3^iii^	2.433 (3)	C2—H2B	0.9700
S1—O3	1.439 (3)	C3—H3A	0.9600
S1—O2	1.447 (3)	C3—H3B	0.9600
S1—O1	1.479 (2)	C3—H3C	0.9600
S1—C5	1.743 (3)	C4—C5	1.359 (4)
O3—Cu1^iv^	2.433 (3)	C4—C6	1.497 (5)
N1—C1	1.318 (4)	C6—H6A	0.9600
N1—C5	1.385 (4)	C6—H6B	0.9600
N2—C1	1.352 (4)	C6—H6C	0.9600
			
N1^i^—Cu1—N1	180.00 (9)	C4—N2—H2C	125.2
N1^i^—Cu1—O1	95.08 (10)	N1—C1—N2	109.4 (3)
N1—Cu1—O1	84.92 (10)	N1—C1—C2	126.5 (3)
N1^i^—Cu1—O1^i^	84.92 (10)	N2—C1—C2	124.1 (3)
N1—Cu1—O1^i^	95.08 (10)	C1—C2—C3	114.7 (3)
O1—Cu1—O1^i^	180.000 (1)	C1—C2—H2A	108.6
N1^i^—Cu1—O3^ii^	88.44 (10)	C3—C2—H2A	108.6
N1—Cu1—O3^ii^	91.56 (10)	C1—C2—H2B	108.6
O1—Cu1—O3^ii^	86.90 (10)	C3—C2—H2B	108.6
O1^i^—Cu1—O3^ii^	93.10 (10)	H2A—C2—H2B	107.6
N1^i^—Cu1—O3^iii^	91.56 (10)	C2—C3—H3A	109.5
N1—Cu1—O3^iii^	88.44 (10)	C2—C3—H3B	109.5
O1—Cu1—O3^iii^	93.10 (10)	H3A—C3—H3B	109.5
O1^i^—Cu1—O3^iii^	86.90 (10)	C2—C3—H3C	109.5
O3^ii^—Cu1—O3^iii^	180.0	H3A—C3—H3C	109.5
O3—S1—O2	112.46 (16)	H3B—C3—H3C	109.5
O3—S1—O1	112.61 (15)	C5—C4—N2	104.3 (3)
O2—S1—O1	113.19 (16)	C5—C4—C6	131.6 (3)
O3—S1—C5	108.32 (16)	N2—C4—C6	124.1 (3)
O2—S1—C5	108.00 (16)	C4—C5—N1	110.2 (3)
O1—S1—C5	101.46 (15)	C4—C5—S1	131.3 (3)
S1—O1—Cu1	118.92 (14)	N1—C5—S1	118.4 (2)
S1—O3—Cu1^iv^	131.33 (15)	C4—C6—H6A	109.5
C1—N1—C5	106.6 (3)	C4—C6—H6B	109.5
C1—N1—Cu1	138.6 (2)	H6A—C6—H6B	109.5
C5—N1—Cu1	114.7 (2)	C4—C6—H6C	109.5
C1—N2—C4	109.5 (3)	H6A—C6—H6C	109.5
C1—N2—H2C	125.2	H6B—C6—H6C	109.5
			
O3—S1—O1—Cu1	128.60 (17)	Cu1—N1—C1—C2	−1.4 (5)
O2—S1—O1—Cu1	−102.44 (19)	C4—N2—C1—N1	−0.5 (4)
C5—S1—O1—Cu1	13.0 (2)	C4—N2—C1—C2	178.2 (3)
N1^i^—Cu1—O1—S1	167.66 (17)	N1—C1—C2—C3	177.2 (4)
N1—Cu1—O1—S1	−12.34 (17)	N2—C1—C2—C3	−1.2 (5)
O3^ii^—Cu1—O1—S1	79.50 (17)	C1—N2—C4—C5	0.3 (4)
O3^iii^—Cu1—O1—S1	−100.50 (17)	C1—N2—C4—C6	−177.9 (3)
O2—S1—O3—Cu1^iv^	180.00 (18)	N2—C4—C5—N1	0.0 (4)
O1—S1—O3—Cu1^iv^	−50.7 (2)	C6—C4—C5—N1	178.0 (3)
C5—S1—O3—Cu1^iv^	60.7 (2)	N2—C4—C5—S1	−176.9 (3)
O1—Cu1—N1—C1	−170.2 (3)	C6—C4—C5—S1	1.1 (6)
O1^i^—Cu1—N1—C1	9.8 (3)	C1—N1—C5—C4	−0.2 (4)
O3^ii^—Cu1—N1—C1	103.0 (3)	Cu1—N1—C5—C4	−177.8 (2)
O3^iii^—Cu1—N1—C1	−77.0 (3)	C1—N1—C5—S1	177.1 (2)
O1—Cu1—N1—C5	6.3 (2)	Cu1—N1—C5—S1	−0.5 (3)
O1^i^—Cu1—N1—C5	−173.7 (2)	O3—S1—C5—C4	50.0 (4)
O3^ii^—Cu1—N1—C5	−80.5 (2)	O2—S1—C5—C4	−72.1 (4)
O3^iii^—Cu1—N1—C5	99.5 (2)	O1—S1—C5—C4	168.7 (3)
C5—N1—C1—N2	0.4 (4)	O3—S1—C5—N1	−126.7 (3)
Cu1—N1—C1—N2	177.1 (2)	O2—S1—C5—N1	111.3 (3)
C5—N1—C1—C2	−178.2 (3)	O1—S1—C5—N1	−8.0 (3)

Symmetry codes: (i) −*x*+1, −*y*+1, −*z*+1; (ii) *x*−1, *y*, *z*; (iii) −*x*+2, −*y*+1, −*z*+1; (iv) *x*+1, *y*, *z*.

### Hydrogen-bond geometry (Å, °)

**Table:**

*D*—H···*A*	*D*—H	H···*A*	*D*···*A*	*D*—H···*A*
N2—H2C···O2^v^	0.86	1.95	2.784 (4)	164.

Symmetry codes: (v) *x*, −*y*+1/2, *z*−1/2.
